# Supplementary material for: Effects of Different Parts on the Chemical Composition, Silage Fermentation Profile, In Vitro and In Situ Digestibility of Paper Mulberry
Source: Animals (Basel). 2021 Feb 5;11(2):413. doi: 10.3390/ani11020413 (PMC7914576; doi:10.3390/ani11020413)
Supplement: Supplementary file 1 [file animals-11-00413-s001.pdf]

Table S1 The components of the buffer solution for *in vitro* experiments

Items[1]	Contents (mL/L)
Water	474
Microelement solution	0.118
Artificial saliva	237
Macroelement solution	237
Resazurin solution	1.18
Reductant solution	47.4

[1] 100 mL microelement solution contains: 13.2 g $CaCl_2 \bullet 2H_2O$, 10.0 g $MnCl_2 \bullet 4H_2O$, 1.0 g $CoCl_2 \bullet 6H_2O$, 8 g $FeCL_2 \bullet 6H_2O$; 100 mL Artificial saliva contains: 3.50 g $NaHCO_3$, 0.40 g NH_4HCO_3; 100 mL Macroelement solution contains: 0.57 g Na_2HPO_4, 0.62 g KH_2PO_4, 0.06 g $MgSO_4 \bullet 7H_2O$; 100 mL Resazurin solution solution contains: 10 g $C_{12}H_7NO_4$; 100 mL Reductant solution contains: 625 mg $Na_2S \bullet 9H_2O$, 0.16 g NaOH.

Table S2 Ingredient and chemical composition of the basal diet fed fistulated cows during the *in vitro* experiment (DM basis)

Ingredients	Amount (g/kg DM)	Ingredients	Amount (g/kg DM)
Whole corn silage	521	Limestone	4.87
Alfalfa	69.31	NaCl	2.58
Soybean meal	79.76	$MgCl_2$	0.87
Corn	38.05	Choline	1.73
Flaked corn	86.78	Premix[1]	3.52
Extrusion soybean	9.76	Water	86.82
Soybean hull	23.14	Total	1000
Cottonseed	29.05	Nutrient levels[2]	
Fatty powder	8.73	Dry matter (g/kg as-fed)	507
Yeast culture	3.24	OM (g/kg DM)	921
Detoxication agent	0.52	NEL (MJ/kg DM)	8.16
$NaHCO_3$	6.43	CP (g/kg DM)	170
$KHCO_3$	3.20	aNDF (g/kg DM)	310
$CaHPO_4$	2.41	ADF (g/kg DM)	203

[1] Premix provided the following per kg of the total mixed ration: Mn 6 000 mg, Fe 4 800 mg, Zn 9 000 mg, Cu 2 600 mg, I 120 mg, Se 80 mg, Co 70 mg, VA 130 000 IU, VD3 36 000 IU, VE 465 IU.

[2] NEL: net energy for lactation. NEL was a calculated value, while the other values were measured.
